# Supplementary material for: Production and Application of Stable Isotope-Labeled Internal Standards for RNA Modification Analysis
Source: Genes (Basel). 2019 Jan 5;10(1):26. doi: 10.3390/genes10010026 (PMC6356711; doi:10.3390/genes10010026)
Supplement: Supplementary file 1 [file genes-10-00026-s001.pdf]

genes

MDPI

Article

Production and Application of Stable Isotope-Labeled Internal Standards for RNA Modification Analysis

Kayla Borland [1], Jan Diesend [2], Taku Ito-Kureha [3], Vigo Heissmeyer [3,4], Christian Hammann [2], Amy H. Buck [5], Stylianos Michalakis [6] and Stefanie Kellner [1,*]

[1] Department of Chemistry, Ludwig Maximilians University Munich, Butenandtstr. 5-13, 81377 Munich, Germany; kaboch@cup.uni-muenchen.de

[2] Department of Life Sciences and Chemistry, Jacobs University Bremen GmbH, Campus Ring 1, 28759 Bremen, Germany; j.diesend@jacobs-university.de (J.D.); c.hammann@jacobs-university.de (C.H.)

[3] Institute for Immunology at the Biomedical Center, Ludwig-Maximilians-Universität München, 82152 Planegg-Martinsried, Germany; Taku.Kureha@med.uni-muenchen.de (T.I.-K.); vigo.heissmeyer@med.uni-muenchen.de (V.H.)

[4] Helmholtz Zentrum München, Research Unit Molecular Immune Regulation, Marchioninistr. 25, 81377 Munich, Germany

[5] Institute of Immunology & Infection and Centre for Immunity, Infection & Evolution, School of Biological Sciences, University of Edinburgh, Edinburgh EH9 3FL, UK; a.buck@ed.ac.uk

[6] Center for Integrated Protein Science Munich CiPSM at the Department of Pharmacy—Center for Drug Research, Ludwig-Maximilians-Universität München, Butenandtstr. 5-13, 81377 Munich, Germany; stylianos.michalakis@cup.uni-muenchen.de

* Correspondence: stefanie.kellner@cup.uni-muenchen.de; Tel.: +49-(0)892-1807-7724

Content:

Figure S1: High-resolution mass spectra of modified nucleosides
Figure S2: Calibration curves
Figure S3: Calculations for absolute quantification of modified nucleosides
Figure S4: Chromatogram of methylated adenosines to determine bacterial contamination in eukaryotic samples
Table S1: Synthetic modified nucleosides and their suppliers
Table S2a: MRM method for detection of nucleosides using the *E. coli* SILIS
Table S2b: MRM method for detection of nucleosides using the yeast SILIS
Table S3a: Mass transition of nucleosides in the *E. coli* SILIS
Table S3b: Mass transition of nucleosides in the yeast SILIS
Table S4: Lower limits of quantification for modified nucleosides in general and for mRNA
Table S5a: Data table from Figure 4
Table S5b: Data table from Figure 5
Table S5c: Data table from Figure 6b

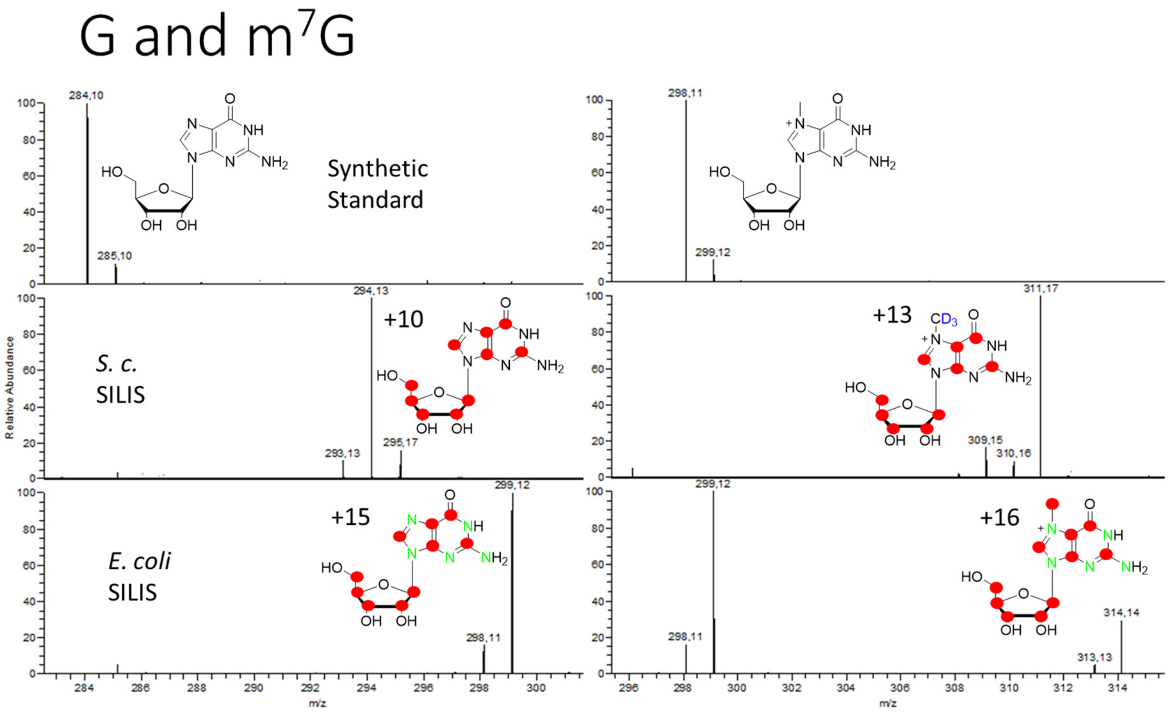

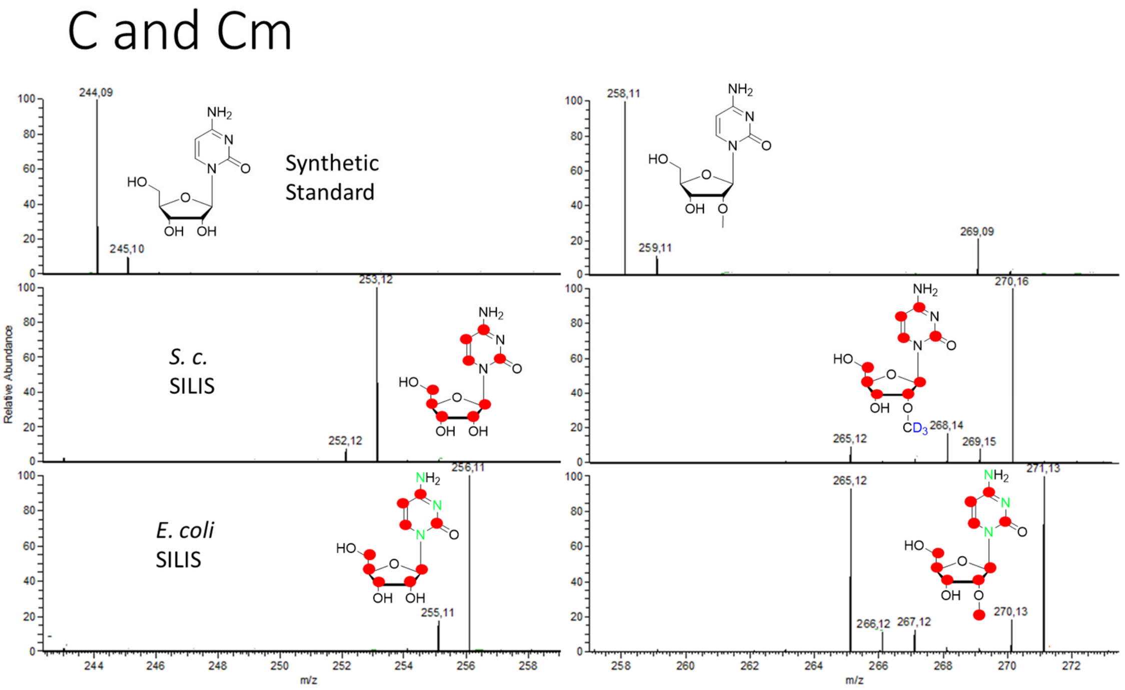

Figure S1. *Cont*

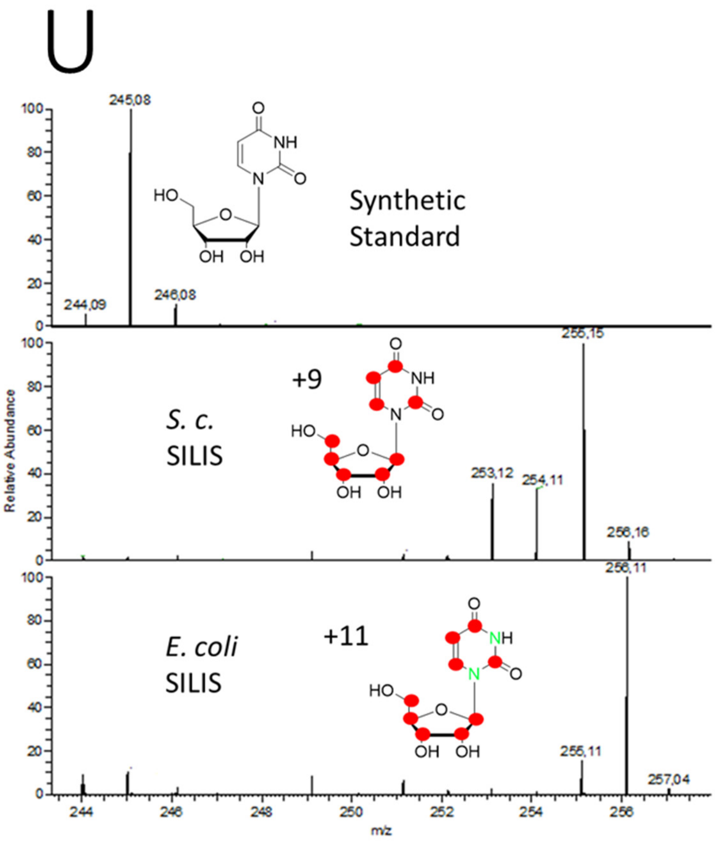

Figure S1: High-resolution mass spectra shows representative nucleosides from the produced labeled RNA digests.

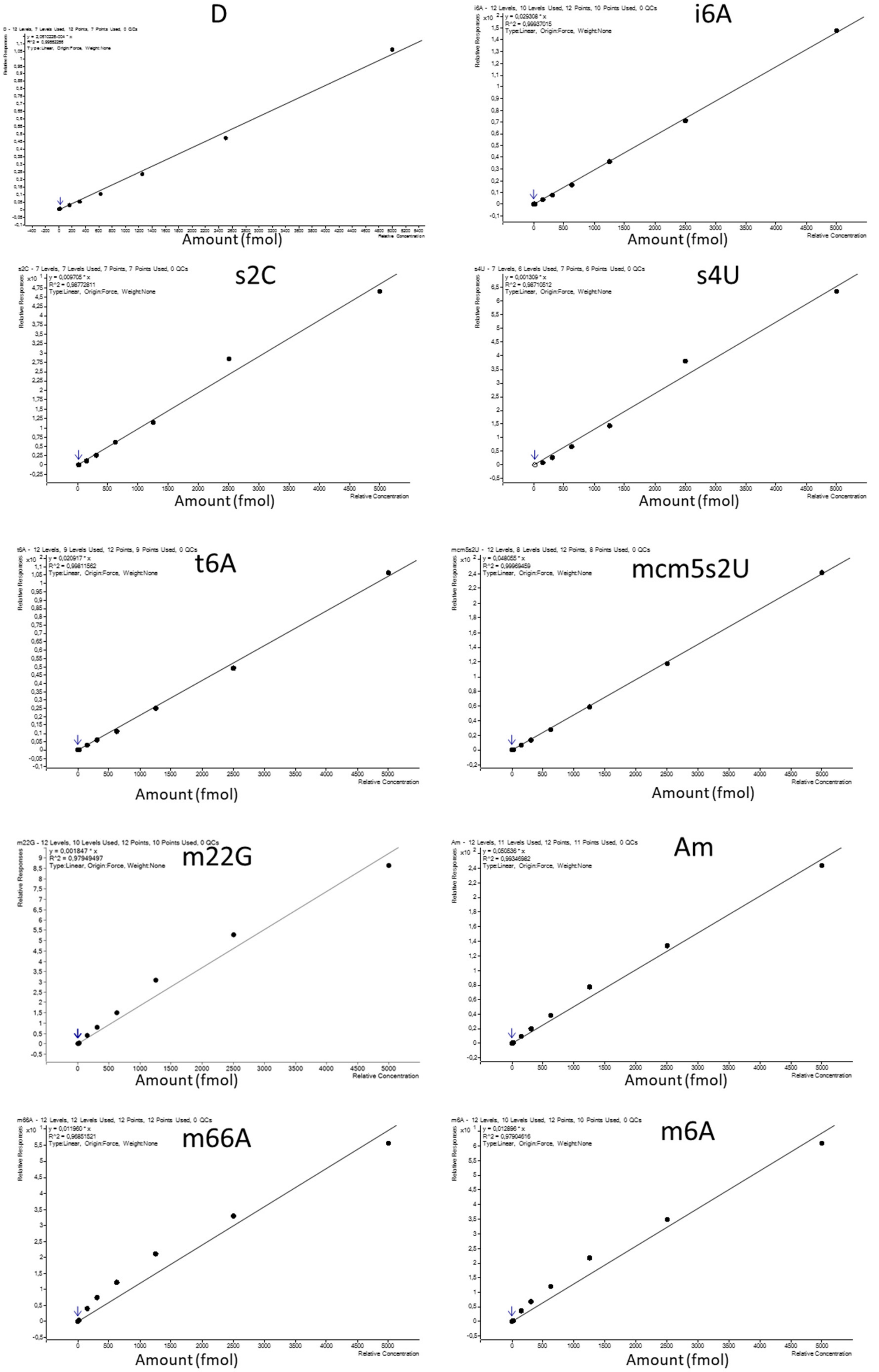

Figure S2. *Cont*

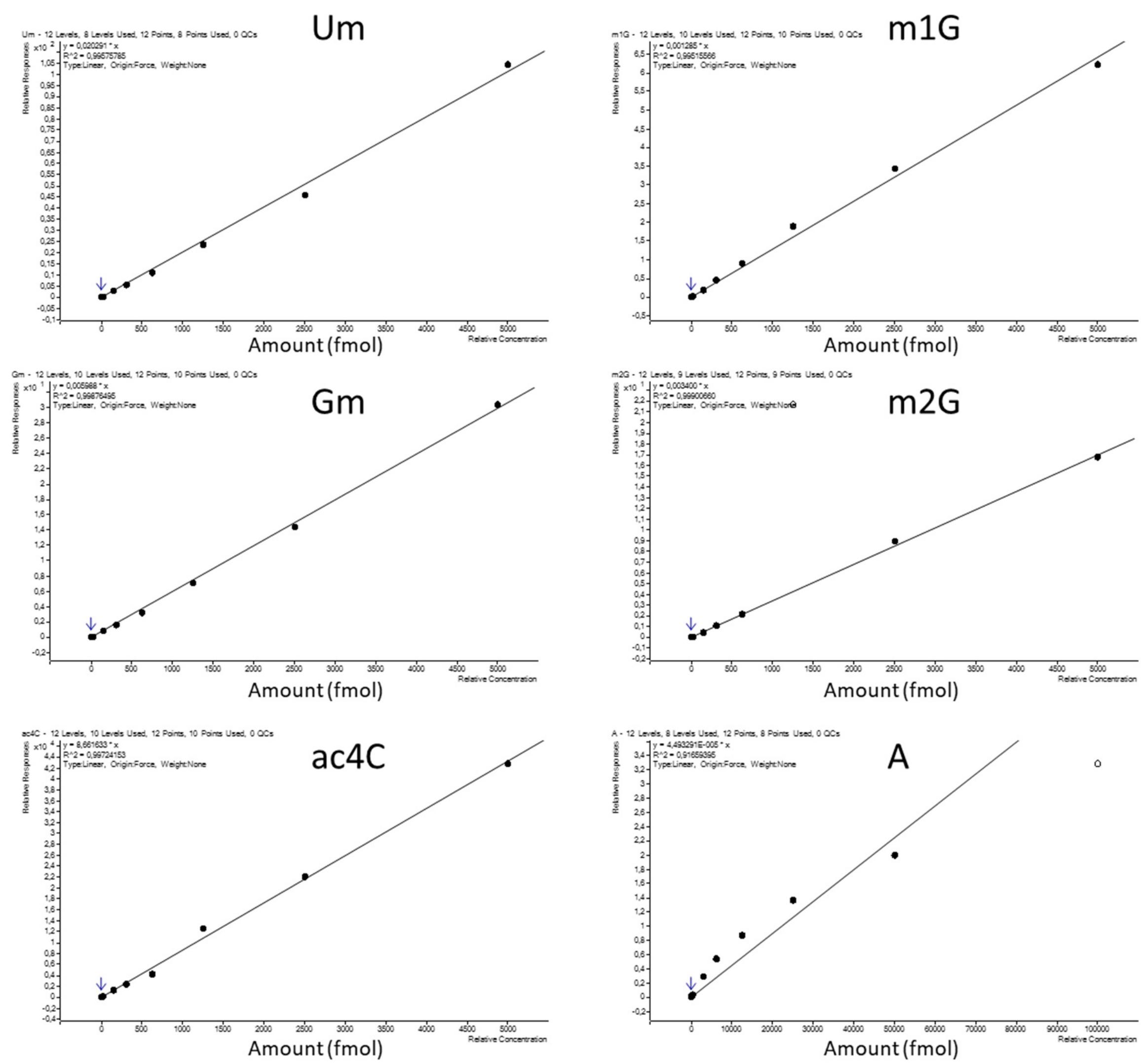

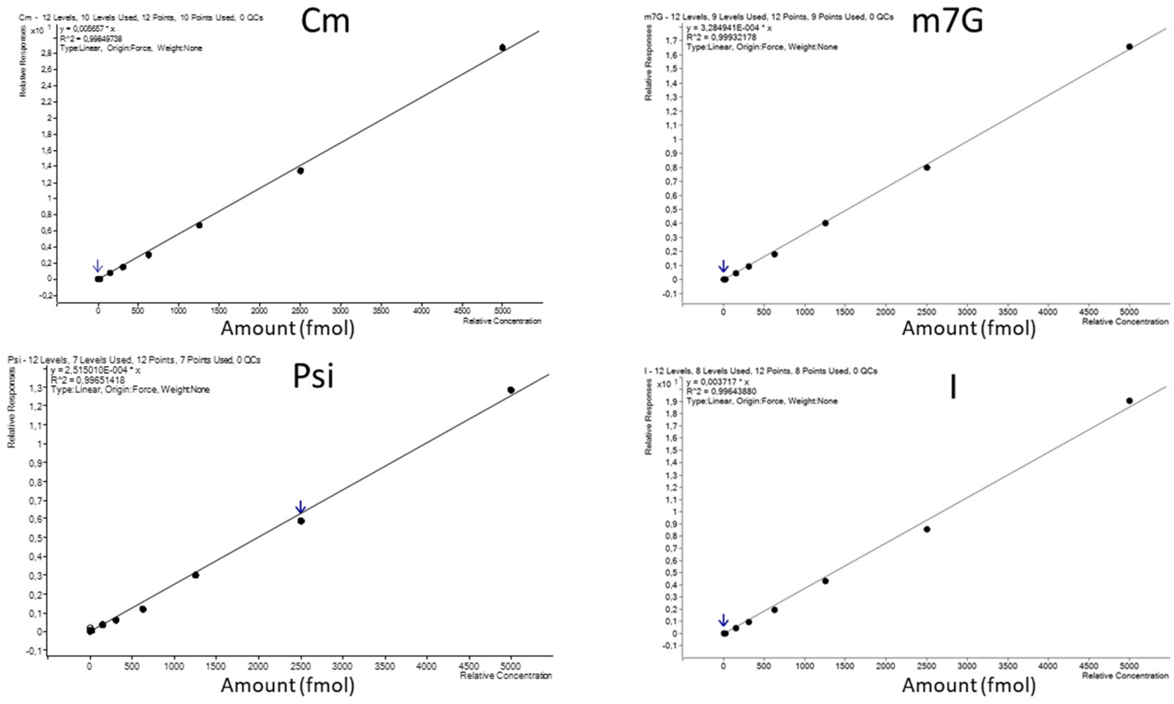

Figure S2. *Cont*

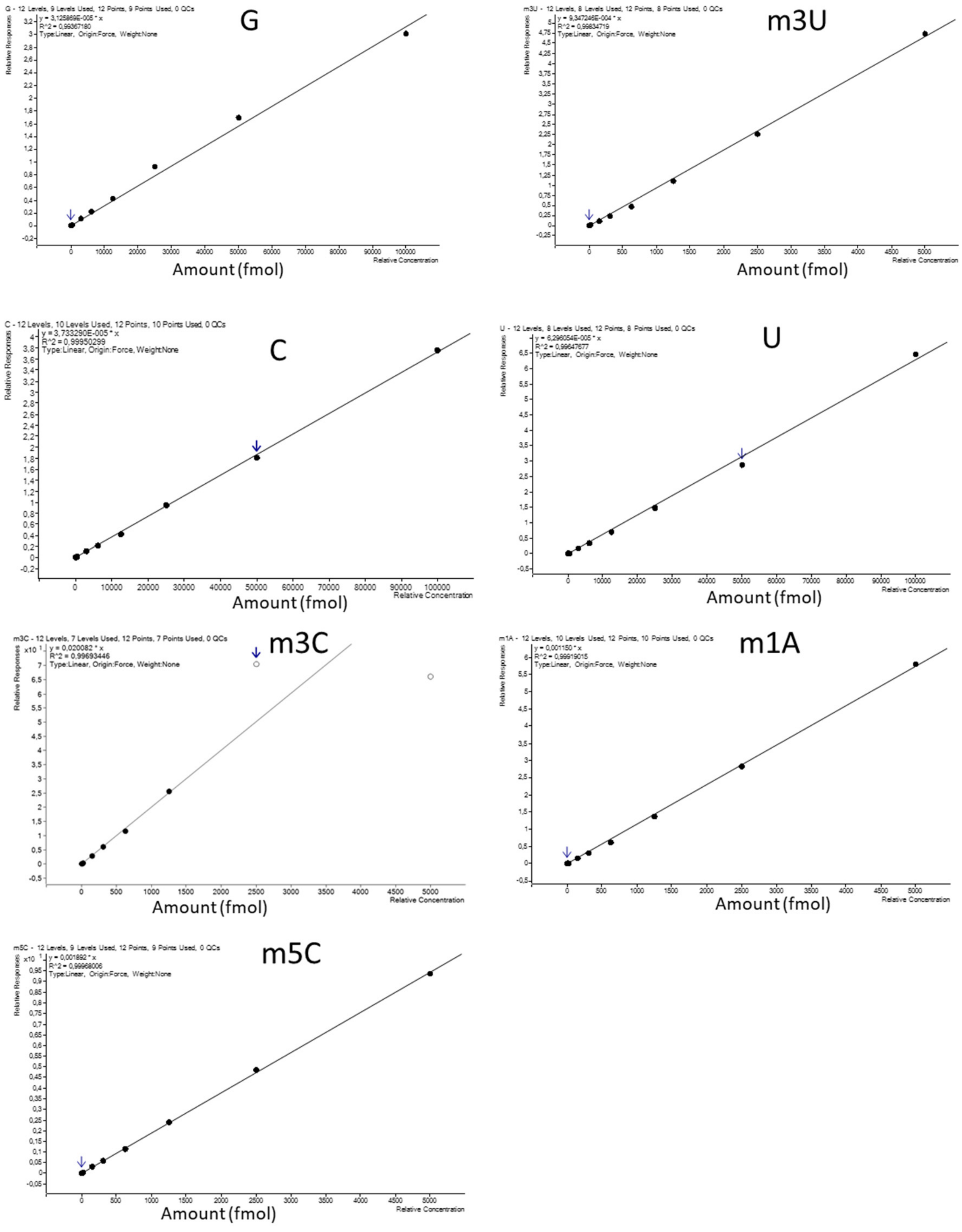

Figure S2: Calibration curves for all compounds in the calibration mix with relative response factor as the y axis and amount in fmol as the x axis. Most calibration curves shown are made with *S.c.* SILIS with the exception of s4U and s2C which were measured with *E. coli* SILIS.

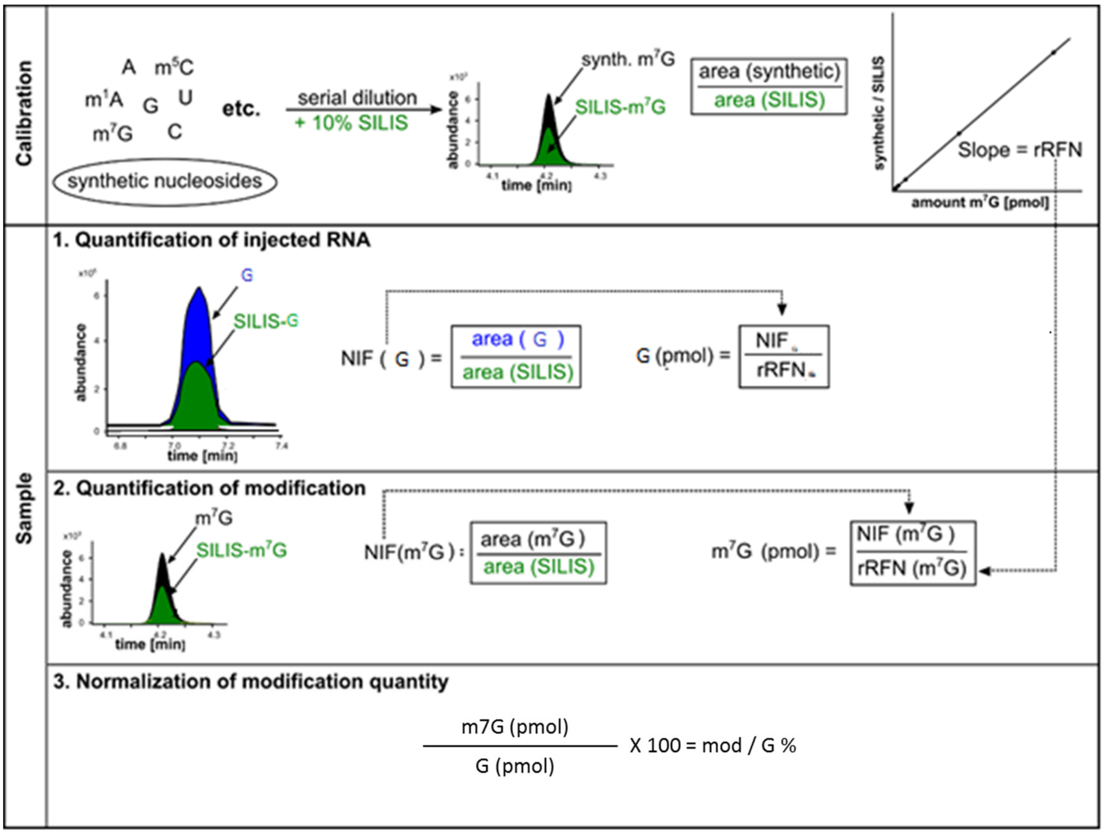

Figure S3: Calculations: The ratio of synthetic standard to SILIS area is plotted over the amount of injected synthetic standard. The slope of the calibration curve is the relative response factor nucleoside (rRFN) and is done for all calibrated nucleosides. The amount of sample RNA injected is quantified by calculation of the nucleoside-isotope-factor (NIF). The NIF is the area of the sample nucleoside peak over the corresponding SILIS peak area. The NIF is then divided by the rRFN to reveal the quantity in pmol.. The amount of modification is reported in percent G; calculated by the pmol of any modification over the pmol of G multiplied by 100.

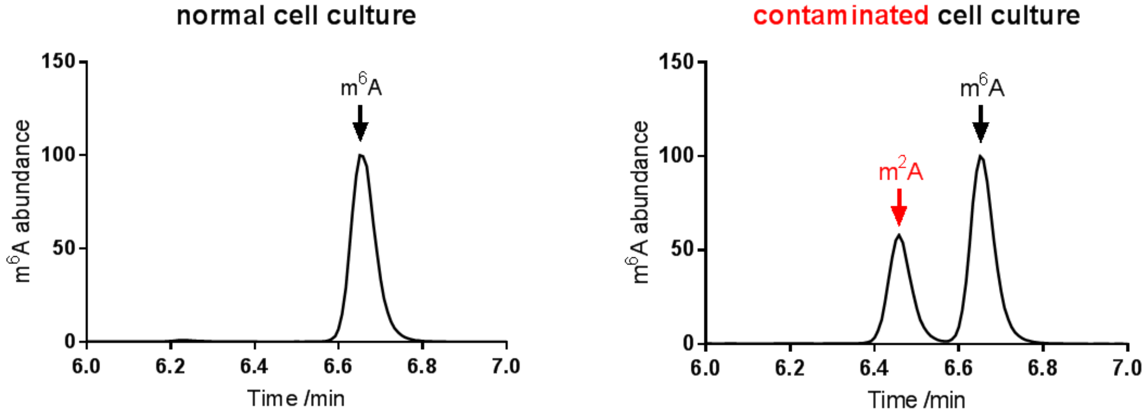

Figure S4: LC-MS/MS trace of the mass transition 282 →150 for methylated adenosines. On the left the chromatogram of a tRNA sample from a normal cell culture is shown. Only 6-methyladenosine (m^6A) can be detected. On the right, there are two peaks eluting. The first one is 2-methyladenosine (m^2A, red), which is a common bacterial tRNA modification and indicates the contamination of the cell culture with bacteria. Later testing revealed that the cell culture sample on the right was contaminated with mycobacteria.

Table S1: Overview of used synthetic standards, their vendors and alternative vendors. Sigma: Sigma, Aldrich, Munich, Germany; Carbosynth, Newbury, UK; TRC: Toronto Research Chemicals, Toronto, CA; Berry & Associates, Dexter, MI, USA.

Nucleoside	Abbreviation	Obtained from:	Also available from:
**cytidine**	C	Sigma	
**2′-O-methylcytidine**	Cm	Carbosynth	
**3-methylcytidine**	m3C	Carbosynth	Carbo and TRC
**5-methylcytidine**	m5C	Carbosynth	
**2-thiocytidine**	s2C	Berry & Associates	
**N4-acetylcytidine**	ac4C	Carbosynth	
**pseudouridine**	Y	Carbosynth	
**uridine**	U	Sigma	
**dihydrouridine**	D	Apollo scientific, UK	
**2′-O-methyluridine**	Um	Carbosynth	
**3-methyluridine**	m3U	Dedon Lab	
**5-methyluridine**	m5U	Carbosynth	
**4-thiouridine**	s4U	ordered from TRC	
**5-methoxycarbonylmethyl-2-thiouridine**	mcm5s2U	Helm Lab	
**guanosine**	G	Sigma	
**1-methylguanosine**	m1G		
**2′-O-methylguanosine**	Gm	Carbosynth	
**N2-methylguanosine**	m2G	Dedon Lab	
**7-methylguanosine**	m7G	Carbosynth	
**N2,N2-dimethylguanosine**	m2,2G	Carbosynth	
**adenosine**	A	Sigma	
**inosine**	I	Carbosynth	
**1-methyladenosine**	m1A		Carbosynth
**2-methyladenosine**	m2A	Dedon Lab	
**2′-O-methyladenosine**	Am	Carbosynth	Carbo and TRC
**N6-methyladenosine**	m6A	Carbosynth	Carbo, TRC and B&A
**N6,N6-dimethyladenosine**	m6,6A	Alfa Chemistry	TRC
**N6-isopentenyladenosine**	i6A	Dedon Lab	TRC
**N6-threonylcarbamoyladenosine**	t6A	TRC	TRC

Table S2a MRM method for sample quantification using the *E. coli* SILIS

Compound Name	Precursor Ion	Product Ion	Ret Time (min)	Delta Ret Time (min)	Fragmentor (V)	Collision Energy (eV)	Cell Accelerator Voltage (V)
**A eSILIS**	283	146	5.1	1	200	40	5
**Am eSILIS**	298	146	6.0	1	110	17	5
**C eSILIS**	256	119	1.9	1	175	30	5
**Cm eSILIS**	271	119	3.7	1	180	9	5
**D eSILIS**	258	121	1.5	1	85	6	5
**G eSILIS**	299	162	4.0	1	95	30	5
**Gm eSILIS**	314	162	4.7	1	100	9	5
**I eSILIS**	283	146	3.8	1	100	9	5
**i6a eSILIS**	356	219	7.9	1.0	140	17	5
**m1G eSILIS**	314	177	4.7	1	105	13	5
**m2G eSILIS**	311	174	5	1	95	17	5
**m3C eSILIS**	271	134	2.5	2	88	14	5
**m3U eSILIS**	271	134	4.2	2	75	9	5
**m5C eSILIS**	271	134	3.4	1	185	13	5
**m5U eSILIS**	271	134	4.0	1	145	10	5
**m66A eSILIS**	313	176	7	1	130	21	5
**m6A eSILIS**	298	161	6.4	1	120	20	5
**m7G eSILIS**	314	177	3.6	2	105	14	5
**Psi eSILIS**	256	218	1.4	1	90	5	5
**s2C eSILIS**	272	135	3.2	2.0	85	13	5
**s4U eSILIS**	272	135	4.5	2	75	17	5
**t6A eSILIS**	434	297	5	2	130	9	5
**U eSILIS**	256	119	2.8	1	95	5	5
**Um eSILIS**	271	119	4.1	2.0	96	8	5
**A**	268	136	5.1	1	200	40	5
**ac4C**	286	154	4.8	1	85	9	5
**Am**	282	136	6.0	1.0	110	17	5
**C**	244	112	1.9	1	175	30	5
**Cm**	258	112	3.7	1	180	9	5
**D**	247	115	1.5	1	85	6	5
**G**	284	152	4.0	1	95	30	5
**Gm**	298	152	4.7	1	100	9	5
**I**	269	137	3.8	1	100	9	5
**i6A**	336	204	7.9	1.0	140	17	5
**m1A**	282	150	2.9	2	110	21	5
**m1G**	298	166	4.7	1	105	13	5
**m22G**	312	180	5.5	1	102	15	5
**m2G**	298	166	5	1	95	17	5

**m3C**	258	126	2.5	2	88	14	5
**m3U**	259	127	4.2	2	75	9	5
**m5C**	258	126	3.4	1	185	13	5
**m5U**	259	127	4.0	1	145	10	5
**m66A**	296	164	7	1	130	21	5
**m6A**	282	150	6.4	1	120	20	5
**m7G**	299	167	3.6	2	105	14	5
**mcm5s2U**	333	201	6	2	92	8	5
**ms2A**	314	182	5	2	125	21	5
**Psi**	245	209	1.4	1	90	5	5
**Q**	410	278	5.8	2	100	11	5
**s2C**	260	128	3.2	2.0	85	13	5
**s4U**	261	129	4.5	2	75	17	5
**t6A**	413	281	5	2	130	9	5
**U**	245	113	2.8	1	95	5	5
**Um**	259	113	4.1	2	96	8	5

Table S2b MRM method for sample quantification using the yeast SILIS

**Compound Name**	**Precursor Ion**	**Product Ion**	**Ret Time (min)**	**Delta Ret Time (min)**	**Fragmentor (V)**	**Collision Energy (eV)**	**Cell Accelerator Voltage (V)**
**A ySILIS**	278	141	5.1	1	200	40	5
**ac4C ySILIS**	297	160	4.8	1	85	9	5
**Am ySILIS**	295	141	6.0	1	110	17	5
**C ySILIS**	253	116	1.9	1	175	30	5
**Cm ySILIS**	270	116	3.7	1	180	9	5
**D ySILIS**	256	119	1.5	1	85	6	5
**G ySILIS**	294	157	4.0	1	95	30	5
**Gm ySILIS**	311	157	4.7	1	100	9	5
**I ySILIS**	279	142	3.8	1	100	9	5
**i6a ySILIS**	351	214	7.9	1	140	17	5
**m1A ySILIS**	295	158	2.9	2	110	21	5
**m1G ySILIS**	311	174	4.7	1	105	13	5
**m22G ySILIS**	328	191	5.5	1	102	15	5
**m2G ySILIS**	311	174	5	1	95	17	5
**m3C ySILIS**	270	133	2.5	2	88	14	5
**m3U ySILIS**	271	134	4.2	2	75	9	5
**m5C ySILIS**	270	133	3.4	1	185	13	5
**m5U ySILIS**	271	134	4.0	1	145	10	5
**m66A ySILIS**	312	175	7	1	130	21	5

**m6A ySILIS**	295	158	6.4	1	120	20	5
**m7G ySILIS**	311	174	3.6	2	105	14	5
**mcm5s2U ySILIS**	347	210	6	2	92	8	5
**Psi ySILIS**	254	218	1.4	1	90	5	5
**Q ySILIS**	427	290	5.8	2	100	11	5
**t6A ySILIS**	428	291	5	2	130	9	5
**U ySILIS**	254	117	2.8	1	95	5	5
**Um ySILIS**	271	117	4.1	2	96	8	5
**A**	268	136	5.1	1	200	40	5
**ac4C**	286	154	4.8	1	85	9	5
**Am**	282	136	6.0	1	110	17	5
**C**	244	112	1.9	1	175	30	5
**Cm**	258	112	3.7	1	180	9	5
**D**	247	115	1.5	1	85	6	5
**G**	284	152	4.0	1	95	30	5
**Gm**	298	152	4.7	1	100	9	5
**I**	269	137	3.8	1	100	9	5
**i6A**	336	204	7.9	1	140	17	5
**m1A**	282	150	2.9	2	110	21	5
**m1G**	298	166	4.7	1	105	13	5
**m22G**	312	180	5.5	1	102	15	5
**m2G**	298	166	5	1	95	17	5
**m3C**	258	126	2.5	2	88	14	5
**m3U**	259	127	4.2	2	75	9	5
**m5C**	258	126	3.4	1	185	13	5
**m5U**	259	127	4.0	1	145	10	5
**m66A**	296	164	7	1	130	21	5
**m6A**	282	150	6.4	1	120	20	5
**m7G**	299	167	3.6	2	105	14	5
**mcm5s2U**	333	201	6	2	92	8	5
**ms2A**	314	182	5	2	125	21	5
**Psi**	245	209	1.4	1	90	5	5
**Q**	410	278	5.8	2	100	11	5
**s2C**	260	128	3.2	2.0	85	13	5
**s4U**	261	129	4.5	2	75	17	5
**t6A**	413	281	5	2	130	9	5
**U**	245	113	2.8	1	95	5	5
**Um**	259	113	4.1	2.0	96	8	5

Table S3a Nucleosides found in the heavy labeled *E. coli* RNA and their mass transitions

Compound	Precursor Ion (m/z)	Product Ion (m/z)
**A eSILIS**	283	146
**Am eSILIS**	298	146
**C eSILIS**	256	119
**Cm eSILIS**	271	119
**D eSILIS**	258	121
**G eSILIS**	299	162
**Gm eSILIS**	314	162
**I eSILIS**	283	146
**i6a eSILIS**	356	219
**m1G eSILIS**	314	177
**m2G eSILIS**	311	174
**m5C eSILIS**	271	134
**m5U eSILIS**	271	134
**m66A eSILIS**	313	176
**m6A eSILIS**	298	161
**m7G eSILIS**	314	177
**Psi eSILIS**	256	218
**s2C eSILIS**	272	135
**s4U eSILIS**	272	135
**t6A eSILIS**	434	297
**U eSILIS**	256	119
**Um eSILIS**	271	119

Table S3b Nucleosides found in the heavy labeled yeast RNA and their mass transitions

Compound	Precursor Ion (m/z)	Product Ion (m/z)
**A ySILIS**	278	141
**ac4C ySILIS**	297	160
**Am ySILIS**	295	141
**C ySILIS**	253	116
**Cm ySILIS**	270	116
**D ySILIS**	256	119
**G ySILIS**	294	157
**Gm ySILIS**	311	157
**I ySILIS**	279	142
**i6a ySILIS**	351	214
**m1A ySILIS**	295	158
**m1G ySILIS**	311	174
**m22G ySILIS**	328	191
**m2G ySILIS**	311	174
**m3C ySILIS**	270	133
**m3U ySILIS**	271	134
**m5C ySILIS**	270	133
**m5U ySILIS**	271	134
**m66A ySILIS**	312	175
**m6A ySILIS**	295	158
**m7G ySILIS**	311	174
**mcm5s2U ySILIS**	347	210
**mcm5U ySILIS**	331	194
**ncm5s2U ySILIS**	329	192
**Psi ySILIS**	254	218
**Q ySILIS**	427	290
**t6A ySILIS**	428	291
**U ySILIS**	254	117
**Um ySILIS**	271	117

Table S4 Absolute lower limits of quantification (fmol) of modified nucleosides (LLOQ) and the LLOQ per injected mRNA (normalized to G)

	**LLOQ**	
	amount (fmol)	LLOQ (fmol)/ average G (fmol) x100
**Psi**	20	0.022853
**D**	3	0.003428
**Cm**	3	0.003428
**m3C**	0.6	0.000686
**m5C**	3	0.003428
**Um**	10	0.011427
**m3U**	20	0.022853
**m5U**	2	0.002285
**s2C**	2	0.002285
**s4U**	3	0.003428
**I**	3	0.003428
**m1A**	0.6	0.000686
**Am**	0.2	0.000686
**m6A**	0.2	0.000229
**ac4C**	0.6	0.000686
**m66A**	0.1	0.000114
**m1G**	0.6	0.000686
**Gm**	3	0.003428
**m2G**	3	0.003428
**m7G**	3	0.003428
**m22G**	0.6	0.000686
**mcm5s2U**	3	0.003428
**i6A**	0.6	0.000686
**t6A**	3	0.003428

genes MDPI

Table S5a: Data table from Figure 4

	E. c. SILIS rep1	*E. c.* SILIS rep2	*E. c.* SILIS rep3	*S. c.* SILIS rep1	*S. c.* SILIS rep2	*S. c.* SILIS rep3	
**Present in both SILISs (Figure 4a)**							
D	10.311	10.871	10.768	10.620	10.019	10.394	
m1G	2.557	2.746	2.818	2.910	2.972	3.127	
m7G	2.287	2.301	2.254	2.389	2.258	2.303	
t6A	1.396	1.304	1.385	1.345	1.257	1.326	
Gm	0.857	0.859	0.821	0.870	0.802	0.803	
I	0.779	0.746	0.755	0.770	0.746	0.767	
Cm	0.285	0.237	0.181	0.537	0.475	0.476	
m6A	0.113	0.119	0.116	0.135	0.125	0.124	
i6A	0.020	0.024	0.025	0.015	0.015	0.016	
Am	0.017	0.019	0.021	0.022	0.023	0.026	
m66A	0.000	0.000	0.001	0.001	0.001	0.001	
**Only present in the *S. c.* SILIS (Figure 4b)**							Specialty
m2G	0.812	0.807	0.767	0.539	0.543	0.536	Reference to m1G
m1A	2.693	3.447	3.470	2.977	3.879	4.583	External cal
m5C	5.313	5.972	5.980	2.681	2.763	2.899	Reference to m5U
m22G	1.571	1.946	1.865	1.820	2.047	2.319	External cal
m5U	2.328	2.149	2.144	1.494	1.471	1.288	External cal
Um	0.754	0.822	0.812	1.732	1.738	1.893	Reference to Cm
m3C	1.843	1.670	1.426	0.893	0.861	0.846	Reference to m5U
ac4C	0.626	0.709	0.622	0.326	0.386	0.292	External cal
mcm5s2U	0.215	0.213	0.169	0.223	0.201	0.191	External cal

Table S5b: Data table from Figure 5

	E. c. LB_1	*E. c.* LB_2	*E. c.* LB_3	*E. c.* M9_1	*E. c.* M9_2	*E. c.* M9_3	*P. a.* M9 1	*P. a.* M9 2	*P. a.* M9 3
Psi	6.672	7.347	7.031	6.076	6.316	6.233	5.588	5.478	5.346
D	8.108	8.383	8.008	8.837	9.223	8.159	5.669	8.304	7.946
m2G	below LLOQ	below LLOQ	below LLOQ	below LLOQ	below LLOQ	below LLOQ	below LLOQ	below LLOQ	below LLOQ
m1A	below LLOQ	below LLOQ	below LLOQ	below LLOQ	below LLOQ	below LLOQ	below LLOQ	below LLOQ	below LLOQ
m5C	below LLOQ	below LLOQ	below LLOQ	below LLOQ	below LLOQ	below LLOQ	below LLOQ	below LLOQ	below LLOQ
m1G	0.650	0.576	0.574	0.610	0.652	0.648	0.705	0.676	0.683
m22G	below LLOQ	below LLOQ	below LLOQ	below LLOQ	below LLOQ	below LLOQ	below LLOQ	below LLOQ	below LLOQ
m7G	2.452	2.374	2.258	2.433	2.368	2.375	3.243	3.137	3.135
m5U	5.114	5.158	4.902	4.942	4.934	4.798	4.855	4.951	4.724
Um	0.376	0.395	0.307	0.243	0.192	0.222	0.134	0.137	0.109
t6A	0.373	0.389	0.313	0.305	0.318	0.307	0.206	0.209	0.216
Gm	0.804	0.826	0.850	0.675	0.692	0.659	0.176	0.176	0.179
m3C	below LLOQ	below LLOQ	below LLOQ	below LLOQ	below LLOQ	below LLOQ	below LLOQ	below LLOQ	below LLOQ
I	0.204	0.215	0.202	0.170	0.178	0.156	0.154	0.145	0.153
Cm	0.057	0.059	0.051	0.049	0.042	0.048	0.080	0.075	0.073
ac4C	0.004	0.008	0.004	0.009	0.008	0.008	below LLOQ	below LLOQ	below LLOQ
mcm5s2U	below LLOQ	below LLOQ	below LLOQ	below LLOQ	below LLOQ	below LLOQ	below LLOQ	below LLOQ	below LLOQ
m6A	0.065	0.132	0.124	0.130	0.128	0.123	0.101	0.102	0.103
i6A	0.002	0.002	0.002	0.002	0.002	0.002	0.005	0.005	0.005
Am	0.017	0.015	0.018	0.016	0.016	0.016	0.030	0.030	0.031
m66A	below LLOQ	below LLOQ	below LLOQ	below LLOQ	below LLOQ	below LLOQ	below LLOQ	below LLOQ	below LLOQ
s4U	0.054	0.626	0.855	1.180	0.315	0.937	1.040	0.811	0.878
s2C	0.167	0.205	0.186	0.194	0.191	0.167	0.208	0.186	0.307

	S. c. 1	*S. c.* 2	*S. c.* 3	*D._d. 1*	*D._d. 2*	*D._d. 3*	*C._e. 1*	*C._e. 2*	*C._e. 3*
Psi	13.661	14.332	12.982	10.260	10.609	10.271	5.061	5.304	6.254
D	14.506	14.224	14.493	10.171	10.959	10.799	2.033	2.301	1.942
m2G	0.305	0.289	0.289	0.224	0.262	0.227	0.051	0.052	0.071
m1A	2.544	2.505	2.543	5.032	4.138	4.582	0.658	0.679	0.669
m5C	1.846	1.786	1.820	1.850	1.938	1.836	0.430	0.441	0.434
m1G	3.517	3.314	3.472	3.079	3.117	3.054	0.598	0.600	0.680
m22G	2.664	2.420	2.589	3.703	3.007	3.544	0.656	0.659	0.644
m7G	1.299	1.272	1.273	3.331	3.454	3.117	0.631	0.638	0.645
m5U	2.572	2.277	2.518	1.372	1.168	1.154	0.682	0.445	0.351
Um	0.255	0.181	0.235	0.966	0.965	0.610	0.866	0.812	0.822
t6A	1.019	0.896	0.931	1.009	0.818	0.784	0.231	0.237	0.260
Gm	0.848	0.781	0.829	1.353	1.410	1.246	0.951	0.932	0.920
m3C	0.306	0.285	0.301	0.727	0.746	0.701	0.068	0.072	0.067
I	0.906	0.856	0.873	0.781	0.773	0.786	0.036	0.038	0.035
Cm	0.695	0.668	0.678	0.669	0.704	0.737	0.477	0.481	0.481
ac4C	0.110	0.105	0.093	0.063	0.067	0.084	0.066	0.084	0.103
mcm5s2U	0.109	0.116	0.110	0.411	0.475	0.425	0.008	0.008	0.007
m6A	0.179	0.146	0.167	0.148	0.117	0.132	0.071	0.071	0.070
i6A	0.104	0.105	0.117	0.047	0.130	0.072	0.001	0.001	0.001
Am	0.113	0.103	0.116	0.355	0.376	0.388	0.960	0.970	0.959
m66A	0.001	0.001	0.001	0.177	0.193	0.120	0.064	0.059	0.056

	HEK 1	HEK 2	HEK 3	*M. m.* brain 1	*M. m.* brain 2	*M. m.* brain 3	*M. m.* liver 1	*M. m.* liver 2	*M. m.* liver 3
Psi	13.284	11.877	13.201	14.710	12.612	13.308	10.433	12.381	16.348
D	10.620	10.019	10.394	10.497	11.656	11.035	10.868	9.753	10.800
m2G	0.539	0.543	0.536	0.594	0.556	0.568	0.624	0.530	0.666
m1A	2.977	3.879	4.583	3.118	3.128	3.041	3.339	2.937	3.247
m5C	2.681	2.763	2.899	2.529	2.495	2.507	2.651	2.333	2.741
m1G	2.910	2.972	3.127	3.255	3.255	3.187	3.383	2.933	3.508
m22G	1.820	2.047	2.319	2.257	2.242	2.197	2.218	2.028	2.229
m7G	2.389	2.258	2.303	1.829	1.895	1.856	1.738	1.543	1.797
m5U	1.494	1.471	1.288	0.674	0.612	0.627	0.648	0.507	0.668
Um	1.732	1.738	1.893	1.437	1.613	1.475	1.208	1.413	1.085
t6A	1.345	1.257	1.326	1.416	1.274	1.332	1.293	1.122	1.304
Gm	0.870	0.802	0.803	0.789	0.854	0.877	0.404	0.489	0.385
m3C	0.893	0.861	0.846	1.051	0.926	0.888	0.812	0.750	0.888
I	0.770	0.746	0.767	0.602	0.612	0.584	0.580	0.497	0.568
Cm	0.537	0.475	0.476	0.582	0.656	0.654	0.602	0.641	0.586
ac4C	0.326	0.386	0.292	0.134	0.080	0.102	0.078	0.065	0.110
mcm5s2U	0.223	0.201	0.191	0.081	0.018	0.092	0.102	0.074	0.086
m6A	0.135	0.125	0.124	0.251	0.255	0.251	0.260	0.247	0.279
i6A	0.015	0.015	0.016	0.048	0.051	0.042	0.064	0.050	0.068
Am	0.022	0.023	0.026	0.037	0.103	0.128	0.106	0.324	0.038
m66A	0.001	0.001	0.001	0.002	0.014	0.010	0.005	0.020	0.002

Table S5c: Data table from Figure 6b

	M. m. **brain 1**	***M. m.*** **brain 2**	***M. m.*** **brain 3**	***M. m.*** **liver 1**	***M. m.*** **liver 2**	***M. m.*** **liver 3**
**m6A**	0.343497	0.374741	0.36192	0.191708	0.229845	0.177888
**m7G**	0.077023	0.11138	0.098379	0.128558	0.083871	0.071753
**Gm**	0.043097	0.054056	0.047701	0.049877	0.055619	0.040185
**Cm**	0.032846	0.030672	0.033159	0.037945	0.087054	0.037659
**m1A**	0.043834	0.02216	0.014301	0.011702	0.025167	0.018145
**Am**	0.021215	0.023379	0.014492	0.028854	0.039273	0.027936
**Um**	0.025887	0.02572	0.025261	0.027112	0.050256	0.027751
**I**	0.02753	0.013562	0.020386	0.01576	0.069667	0.016231
**m2G**	0.04162	0.01978	0.01472	0.004941	0.019116	0.013516
**m5U**	0.013019	0.013915	0.024981	0.093623	0.195699	0.023944
**m1G**	0.012174	0.005405	0.004853	below LLOQ	0	0.00577
**m66A**	0.000792	0.000803	0.000555	0.000437	0.001041	0.000544
**m22G**	0.00444	0.00227	0.002168	below LLOQ	below LLOQ	below LLOQ
**m5C**	0.009534	0.006	0.005234	0.007464	below LLOQ	0.007353
**t6A**	0.005305	0.00367	0.005382	0.004546	0.006739	0.00271
**m3C**	0.004104	0.001624	below LLOQ	0.007423	0.024755	0.012224
